# A Diabetic Retinopathy Screening Tool for Low-Income Adults in Mexico

**DOI:** 10.5888/pcd14.170157

**Published:** 2017-10-12

**Authors:** Kenny Mendoza-Herrera, Amado D. Quezada, Andrea Pedroza-Tobías, Cesar Hernández-Alcaraz, Jans Fromow-Guerra, Simón Barquera

**Affiliations:** 1 Center for Nutrition and Health Research, National Institute of Public Health, Cuernavaca, Morelos, México; 2 Center for Evaluation and Surveys Research, National Institute of Public Health, Cuernavaca, Morelos, México; 3 Association for the Prevention of Blindness in Mexico, México City, México

## Abstract

**Introduction:**

A national diabetic retinopathy screening program does not exist in Mexico as of 2017. Our objective was to develop a screening tool based on a predictive model for early detection of diabetic retinopathy in a low-income population.

**Methods:**

We analyzed biochemical, clinical, anthropometric, and sociodemographic information from 1,000 adults with diabetes in low-income communities in Mexico (from 11,468 adults recruited in 2014–2016). A comprehensive ophthalmologic evaluation was performed. We developed the screening tool through the following stages: 1) development of a theoretical predictive model, 2) performance assessment and validation of the model using cross-validation and the area under the receiver operating characteristic curve (AUC ROC), and 3) optimization of cut points for the classification of diabetic retinopathy. We identified points along the AUC ROC that minimized the misclassification cost function and considered various scenarios of misclassification costs and diabetic retinopathy prevalence.

**Results:**

Time since diabetes diagnosis, high blood glucose levels, systolic hypertension, and physical inactivity were considered risk factors in our screening tool. The mean AUC ROC of our model was 0.780 (validation data set). The optimized cut point that best represented our study population (*z *= −0.640) had a sensitivity of 82.9% and a specificity of 61.9%.

**Conclusion:**

We developed a low-cost and easy-to-apply screening tool to detect people at high risk of diabetic retinopathy in Mexico. Although classification performance of our tool was acceptable (AUC ROC > 0.75), error rates (precision) depend on false-negative and false-positive rates. Therefore, confirmatory assessment of all cases is mandatory.

**Editor’s Note:** This article is the winner of *PCD*‘s 2017 Student Research Paper Contest in the Graduate category.

## Introduction

In 2016, diabetes was declared a national epidemiologic emergency in Mexico (1). In 2006, the estimated prevalence of diabetes in Mexican adults was 14.4% (2). Mortality rates attributable to this disease in Mexico are among the highest in the world (3). By 2012, 74.7% of Mexican adults with diagnosed diabetes had inadequate glycemic control (4). Diabetes is associated with the development and progression of diabetic retinopathy (5–8), a major cause of sight loss and blindness in Latin American countries (9). A population-based survey from 2010 in the state of Chiapas found that 38.9% of adults aged 50 or older with diabetes had diabetic retinopathy and 21.0% had proliferative diabetic retinopathy (10).

Long-term diabetes and hypertension are consistently associated with diabetic retinopathy (5–8,11–13). The Mexican National Nutrition Survey 2006 found that the mean time since diabetes diagnosis among adults was more than 8 years (2). In 2012, an estimated 65.6% of adults with diabetes had hypertension (14). In this context, an epidemic of diabetes complications, including diabetic retinopathy, could worsen in Mexico, and the study of screening systems for diabetic retinopathy is important.

Diabetic retinopathy ranks third in direct costs generated by diabetes complications in Mexico (15); these costs result from specialized procedures for diagnosis and treatment. A cost-benefit analysis to identify optimal cut points for identifying people who are at risk for diabetic retinopathy and who need a comprehensive ophthalmologic evaluation is an approach to developing an adequate-performance screening tool (16); however, such an approach would be complex because of the detailed cost information required.

Our objective was to develop a practical screening tool based on a predictive model and a simplification of a cost-benefit analysis to optimize cut points for early detection of diabetic retinopathy in low-income communities in Mexico.

## Methods

We conducted a screening protocol for eye-related complications of diabetes from May 1, 2014, to June 30, 2016, in 3 low-income municipalities in the state of Morelos. We recruited 11,468 adults (aged ≥20 y) for a screening of chronic diseases in our mobile unit and community health centers. From these participants, we invited those with a type 2 diabetes diagnosis (n = 1,768 [15.4%]) to a comprehensive ophthalmologic evaluation. Exclusion criteria for this evaluation were signs of ocular infection or pregnancy.

Of the 1,768 participants, 538 declined to participate in the ophthalmologic evaluation, 1 person was excluded because the quality of photographs was not adequate for grading, and 229 participants did not have a photographic assessment at the time of analysis. One thousand participants (56.6%) completed the procedure. We obtained informed consent from all participants, and the protocol was approved by the ethics, research, and biosecurity committees of the Mexican National Institute of Public Health.

### Data collection and definition of variables

All participants had at least 1 glycemic assessment (fasting [≥8 h] capillary or random capillary glycemia [glucometer method] or fasting venous glycemia [glucose oxidase method]). Fasting serum triglycerides, total cholesterol, and high-density lipoprotein cholesterol were assessed by enzymatic method (n = 418) and serum insulin with radioimmunoassay method (n = 112) for a portion of the sample; because of logistical and budgetary constraints, the entire sample could not be assessed for these variables.

High blood glucose was defined as a fasting glucose of 126 mg/dL or more or, if fasting glucose was unavailable, as random glucose of 200 mg/dL or more (17). Insulin resistance was classified by using a homeostasis model assessment value of 3.8 or more (18).

Hypertriglyceridemia was defined as triglycerides of 150 mg/dL or more, hypercholesterolemia as total cholesterol of 200 mg/dL or more, and hypoalphalipoproteinemia as high-density lipoprotein cholesterol of less than 50 mg/dL for women and less than 40 mg/dL for men (19,20).

Blood pressure was measured twice (interval of 30 seconds). We diagnosed high systolic/diastolic blood pressure when the average of the assessments was ≥140/≥90 mm Hg. Likewise, we recorded whether participants reported a diagnosis of hypertension (21).

Weight, height, and waist circumference were measured by trained personnel using standard protocols. Body mass index (BMI; weight in kilograms divided by height in m^2^ [kg/m^2^]) was calculated: overweight was defined as a BMI of 25.0 to 29.9 and obesity as a BMI of 30.0 or more (22). Abdominal obesity was defined as a waist circumference of 80 cm or more for women and 90 cm or more for men (19).

Data on sociodemographic characteristics and clinical history were collected by trained interviewers through an adapted version of the questionnaires applied in the National Health and Nutrition Survey of Mexico (23). We used the time since diabetes diagnosis as a proxy of duration of type 2 diabetes and categorized it into 4 intervals (<5 y, 5 y to <10 y, 10 y to <15 y and ≥15 y). Participants reported whether they followed diet and physical activity recommendations to control their diabetes.

We conducted a principal component analysis of 15 characteristics related to household appliances and services (eg, ownership of car, telephone, computer, vacuum cleaner, washing machine, refrigerator, pay television, internet) as a proxy for socioeconomic status (SES). Similar methods have been used (14). These characteristics had a factorial loading of 0.30 or more. The first principal component was divided into tertiles and used as a proxy for low SES, medium SES, and high SES.

### Ophthalmologic evaluation

All participants were interviewed by using a validated questionnaire for ocular assessment. The following data were collected by trained technicians: best-corrected visual acuity, refractometry (by using an automated refractor [Huvitz HRK-7000]), and intraocular pressure (by using a rebound tonometer [Icare TA01i]). Afterwards, all participants received a photographic evaluation of their posterior pole (45° nonmydriatic fundus camera [DRS-Centervue]). Participants were dilated with tropicamide only if the quality of the photographs was not adequate for grading. We took 3 fields of the posterior pole using a standardized protocol. The first field centered on the optic nerve, the second field centered on the fovea, and third field was temporal to the macula but included the fovea. This protocol has an adequate level of sensitivity and specificity for grading referable stages of diabetic retinopathy (24).

All photographs were sent to Eye Knowledge Network (www.eyeknowledge.net). All cases were masked and reviewed by trained graders from the Hospital Luis Sánchez Bulnes of the Association for the Prevention of Blindness in Mexico. The cases were graded by using the Revised English Diabetic Eye Screening Program Grading System (25), which allows prompt referral of proliferative stages of diabetic retinopathy and macular edema. Diabetic retinopathy was recorded when a participant had background diabetic retinopathy, preproliferative diabetic retinopathy, or proliferative diabetic retinopathy.

### Statistical analysis

We tabulated categorical variables as frequency and proportion distributions and quantitative variables as measures of central tendency (mean or median) and dispersion (standard deviation [SD] or interquartile range). We set statistical significance at an α of .05. We compared measures of central tendency according to diabetic retinopathy status of participants (has diabetic retinopathy or does not have diabetic retinopathy) by using the Student *t* test or Mann–Whitney *U* test, depending on the distribution of the quantitative variables. We used a χ^2 ^test or Fisher exact test to compare the prevalence of diabetic retinopathy across categories of nonquantitative variables. We conducted a descriptive analysis to compare sociodemographic and clinical characteristics and diabetic retinopathy risk factors between participants and nonparticipants.

We developed the screening tool in 3 stages: 1) we developed the theoretical predictive model, 2) we assessed the performance of the model and conducted a validation analysis, and 3) we optimized risk-score cut points for diabetic retinopathy classification.

#### Development of the theoretical predictive model

For multivariate analysis, we included only participants who had complete information on diabetic retinopathy status (the dependent variable), and we determined whether at least 95% of the participants provided information for each of the independent variables. If 5% or more of the participants did not provide information for an independent variable (theoretical risk factors of diabetic retinopathy), we used multiple imputation through a logistic regression model, where diabetic retinopathy, sex, age, and self-reported diabetes screening were the independent variables, to complete the information.

We generated a predictive probit model based on theoretical risk factors of diabetic retinopathy (5–8,11–13). We decided to use this model to develop our tool because of its easy interpretability as a *z* score from its linear equation and because it provides a predicted probability for the linear predictor (applying the standard normal cumulative function). Familiarity with this distribution provides a better understanding of coefficients and predicted *z* scores. The dependent variable was diabetic retinopathy, and the 4 predictors were time since diabetes diagnosis, high blood glucose, high systolic blood pressure, and physical inactivity. We estimated probabilities adjusted by covariables of having diabetic retinopathy given each risk factor category though predictive margins.

#### Performance assessment and validation

We used the *k*-fold cross-validation method (*k *= 10 partitions) and the area under the receiver operating characteristic curve (AUC ROC). To assess the performance of the model in training and validation data sets, we randomly divided the sample into 10 partitions. In each partition, one segment was reserved for model validation (validation data set, n ~ 10%), while the rest of the sample in this partition was used as a training subsample (training data set, n ~ 90%). We calculated the AUC ROC for each iteration and its mean for the 10 iterations.

#### Optimization of risk-score cut points for diabetic retinopathy classification

We developed a risk score for diabetic retinopathy based on the *z* predictor of our statistical model. In this way, the attributable score of each risk factor was equivalent to its probit coefficient.

The use of a cost-benefit analysis to select cut points implies knowledge of true and false classification costs; however, it is difficult to have such complete information. To select the optimal cut points of the *z* predictor to classify diabetic retinopathy, we decided to focus on misclassification costs only through the misclassification cost term (16). We identified points along the ROC curve that minimized the misclassification cost function for various scenarios of misclassification costs and diabetic retinopathy prevalence. The costs of true classification were assumed as null, and the examples of the variations of misclassification ratios were set according to consequences in health costs of screening for diabetic retinopathy.

We estimated sensitivity and specificity across AUC ROC and isocost curves, which minimized the costs of misclassification. Likewise, we estimated positive predictive values and negative predictive values.

We considered the following scenarios for the optimization of the cut points: diabetic retinopathy prevalence of 35.0%, 40.0%, and 45.0%, and the observed prevalence in our sample. We examined various ratios of cost misclassification (classification costs of false negatives divided by classification costs of false positives). We examined ratios of 1, 4, and 10, assuming that classification of a false negative would generate higher health care costs than would classification of a false positive.

The statistical analysis was conducted by using Stata version 13.1 (StataCorp LLC) and RStudio version 1.0.136 with the OptimalCutpoints package.

## Results

The mean age of our sample was 57.2 y (SD, 11.0 y), and 73.0% were women. The prevalence of diabetic retinopathy was 31.7% (Table 1); 18.9% had background diabetic retinopathy, 5.7% had preproliferative diabetic retinopathy, and 7.1% of participants had active proliferative diabetic retinopathy.

**Table 1:** Prevalence of Diabetic Retinopathy^a^ by Sociodemographic and Clinical Characteristics, and Means/Medians for Other Clinical Characteristics by Diabetic Retinopathy^a^ Status of Study Population in 3 Low-Income Municipalities, Mexico, 2014–2016

Characteristics	Total (N = 1,000)	Has Diabetic Retinopathy, % (n = 317)	Does Not Have Diabetic Retinopathy, % (n = 683)	*P* Value^b^
**Overall**	1,000	31.7	68.3	
**Sex^c^**				
Female	730	30.6	69.4	.20
Male	270	34.8	65.2	
**Socioeconomic status^c^^,^ d**				
Low	332	35.5	64.5	.04
Middle	332	32.8	67.2	
High	331	26.6	73.4	
**Marital status^c^**				
Single	100	20.0	80.0	.01
Married	675	31.6	68.4	
Divorced	77	41.6	58.4	
Widowed	133	35.3	64.7	
**Can speak an indigenous language^c^**				
Yes	47	34.0	66.0	.71
No	949	31.5	68.5	
**Education^c^**				
None	162	34.6	65.4	.06
Some elementary school	454	33.5	66.5	
Some junior high school	237	32.9	67.1	
Some high school	82	23.2	76.8	
Some bachelor’s degree or more	63	19.1	80.9	
**Health system affiliation^c^**				
None	83	30.1	69.9	.26
IMSS	150	27.3	72.7	
ISSSTE	72	23.6	76.4	
Seguro Popular	681	33.5	66.5	
Private	13	46.2	53.8	
Other	1	0.0	100.0	
**Body mass index,^c^ kg/m^2^**				
<25.0	247	44.9	55.1	<.001
25.0–29.9	416	30.8	69.2	
≥30.0	321	23.1	76.9	
**Abdominal obesity (waist circumference ≥80 cm for women and ≥90 cm for men)^c^**				
Yes	869	30.4	69.6	.008
No	115	42.6	57.4	
**Triglycerides ≥150 mg/dL^e^**				
Yes	294	34.0	66.0	.32
No	124	29.0	70.1	
**Cholesterol ≥200 mg/dL^e^**				
Yes	168	37.5	62.5	.08
No	250	29.2	70.8	
**High-density lipoprotein cholesterol <50 mg/dL for women and <40 mg/dL for men^e^**				
Yes	329	31.3	68.7	.30
No	89	37.1	62.9	
**Insulin resistance HOMA index ≥3.8^f^**				
Yes	48	39.6	60.4	.004
No	64	15.6	84.4	
**High blood glucose^c^ (fasting glucose ≥126 mg/dL or random glucose ≥200 mg/dL)**				
Yes	603	38.1	61.9	<.001
No	345	20.0	80.0	
**General hypertension^c^ (previous diagnosis or measurement of blood pressure ≥140/≥90 mm Hg)**				
Yes	524	35.5	64.5	.006
No	469	27.3	72.7	
**Physical activity used to control diabetes^g^^,^ h**				
Yes	272	26.8	73.2	.01
No	554	35.6	64.4	
**Diet used to control diabetes^g^^,^ h**				
Yes	345	30.4	69.6	.23
No	483	34.4	65.6	
**Age, mean (SD), y^c^**	57.2 (11.0)	57.9 (9.3)	56.9 (11.7)	.16
**Time since diabetes diagnosis, median (IQR), y^c^**	7.0 (3.0–14.0)	13.0 (8.0–18.0)	5.0 (2.0–10.0)	<.001
**Fasting capillary glucose, median (IQR), mg/dL^i^**	149.0 (118.0–221.0)	194.5 (140.0–243.0)	137.0 (113.0–195.0)	<.001
**Random capillary glucose, median (IQR), mg/dL^j^**	214.5 (155.0- 295.0)	240.0 (182.0–325.0)	196.0 (148.0–273.0)	<.001
**Fasting venous glucose, median (IQR), mg/dL^k^**	153.0 (117.0–219.0)	198.0 (146.0–252.0)	135.5 (110.0–197.0)	<.001
**Insulin, median (IQR), µIU/mL^f^**	9.75 (6.7–13.8)	10.4 (7.3–15.6)	9.5 (6.6–13.7)	.48
**Systolic blood pressure, median (IQR), mm Hg^c^**	127.5 (115.5–142.0)	131.5 (118.5–147.5)	126.5 (114.0–140.0)	<.001
**Diastolic blood pressure, median (IQR), mm Hg^c^**	72.0 (64.0–79.5)	72.5 (65.0–80.5)	71.5 (63.5–79.5)	.19

Abbreviations: HOMA, homeostasis model assessment; IMSS, the Mexican Social Security Institute (Spanish: Instituto Mexicano del Seguro Social); IQR, interquartile range; ISSSTE, the Institute for Social Security and Services for State Workers (Spanish: Instituto de Seguridad y Servicios Sociales de los Trabajadores del Estado).

a Diabetic retinopathy classification according to Revised English Diabetic Eye Screening Program Grading System (grade 1, grade 2, or grade 3) (25).

b χ^2^ test (contingency tables for more than 2 categories or proportion comparison), Student *t* test, or Mann–Whitney *U* test.

c The percentage of participants with missing data was <5.0% or with complete information.

d Socioeconomic index developed by using first principal component methodology.

e Prevalence of diabetic retinopathy was 32.5% among those measured for triglycerides, total cholesterol, and high-density lipoprotein cholesterol (n *=* 418).

f Prevalence of diabetic retinopathy was 25.9% among those measured for insulin (n = 112).

g The percentage of participants with missing data ≥5.0%.

h Determined by answer to question “Do you have any other treatment for sugar control?” Exercise (no/yes) and diet (yes/no) were provided as possible responses.

i Prevalence of diabetic retinopathy was 30.7% among those measured for fasting capillary glucose (n = 423).

j Prevalence of diabetic retinopathy was 31.6% among those measured for random capillary glucose (n = 402).

k Prevalence of diabetic retinopathy was 32.5% among those measured for fasting venous glucose (n = 418).

The prevalence of diabetic retinopathy was significantly higher among participants with insulin resistance, high blood glucose, and hypertension than among participants without those conditions. Participants with diabetic retinopathy had significantly longer times since diabetes diagnosis, higher blood glucose levels, and higher systolic blood pressure than those without diabetic retinopathy. In contrast, the prevalence of diabetic retinopathy was lower among participants who were overweight or obese, had abdominal obesity, or used physical activity to control their diabetes than among participants without these characteristics. The prevalence of diabetic retinopathy was highest, by SES, in the lowest tertile of SES and highest, by marital status, among divorced adults (Table 1).

We found no significant differences in the distribution of sociodemographic characteristics, clinical characteristics, or diabetic retinopathy risk factors between participants and nonparticipants.

### Development and cross-validation of predictive model

From all independent variables included in our model, except physical activity (data were missing for 17.0% of participants), had at least 95.0% of information. After multiple imputation analysis for physical activity, we obtained a probit model with 939 observations.

According to our multivariate analysis (Table 2), time since diabetes diagnosis was positively associated with the estimated probability of diabetic retinopathy. For example, the probability of diabetic retinopathy was 11.4% (95% confidence interval [CI], 7.9%–14.9%) when time since diabetes diagnosis was less than 5 years, whereas the probability was 56.0% (95% CI, 49.5%–62.6%) when time since diabetes diagnosis was 15 years or more. Similarly, the probability of diabetic retinopathy was higher among those with high blood glucose (35.6%) and high systolic blood pressure (37.4%) than among those without those conditions (23.9% and 29.3%, respectively). On the other hand, participants who reported using physical activity to control diabetes had a lower predicted probability of diabetic retinopathy (25.4%) than those who reported not using physical activity (34.8%).

**Table 2:** Predictive Multivariate Model in the Development of a Screening Tool for Diabetic Retinopathy for Use in Low-Income Communities, Mexico, 2014–2016

Risk Factors for Diabetic Retinopathy	Predictive Probit Model (n = 939)^a^			
	Coefficient (SE)	*P* Value^b^	Estimated Probability^c^, % (95% CI)	*P* Value^b^
**Time since diabetes diagnosis, y**				
<5	—d	—d	11.4 (7.9–14.9)	—d
5 to <10	0.55 (0.13)	<.001	24.9 (19.2–30.6)	<.001
10 to <15	1.16 (0.14)	<.001	46.6 (39.4–53.9)	<.001
≥15	1.41 (0.13)	<.001	56.0 (49.5–62.6)	<.001
**High blood glucose (fasting venous or capillary glucose ≥126 mg/dL or random capillary glucose ≥200 mg/dL)**				
No	—d	—d	23.9 (19.5–28.3)	—d
Yes	0.41 (0.10)	<.001	35.6 (32.2–39.0)	<.001
**High systolic blood pressure (≥140 mm Hg)**				
No	—d	—d	29.3 (26.2–32.4)	—d
Yes	0.27 (0.10)	.007	37.4 (32.3–42.5)	.007
**Physical activity used to control diabetes^e^**				
No	—d	—d	34.8 (31.4–38.2)	—d
Yes	−0.33 (0.11)	.002	25.4 (20.9–30.0)	.002
**Constant**	−1.48 (0.12)	<.001	— d	—d

Abbreviations: CI, confidence interval; SE, standard error.

a Multivariate probit model with any grade of diabetic retinopathy (grade 1, grade 2, or grade 3) as dependent variable according to Revised English Diabetic Eye Screening Program Grading System (25).

b *P* value for probit coefficients or for comparison of estimated probabilities among categories and lowest category of different variables.

c Obtained by predictive margins.

d Lowest category or estimated probability of constant.

e Determined by answer to question “Do you have any other treatment for sugar control?” Exercise (no/yes) was provided as a possible response.

According to the cross-validation analysis (Table 3), the diagnostic performance of our model was similar between training data sets (mean AUC ROC = 0.780) and validation data sets (mean AUC ROC = 0.778).

**Table 3:** Cross-Validation Analysis (*k* = 10) of Predictive Probit Model (n = 939) in the Development of a Screening Tool for Diabetic Retinopathy for Use in Low-Income Communities, Mexico, 2014–2016

Iteration	Training Data Set (n ~ 90%), AUC ROC (95% CI)	Validation Data Set (n ~ 10%), AUC ROC (95% CI)
1	0.775 (0.742–0.809)	0.806 (0.720–0.891)
2	0.780 (0.747–0.813)	0.784 (0.690–0.877)
3	0.783 (0.751–0.815)	0.756 (0.642–0.870)
4	0.782 (0.750–0.814)	0.764 (0.659–0.869)
5	0.777 (0.744–0.810)	0.806 (0.712–0.899)
6	0.779 (0.747–0.811)	0.780 (0.664–0.896)
7	0.786 (0.754–0.818)	0.723 (0.603–0.842)
8	0.783 (0.750–0.815)	0.754 (0.653–0.855)
9	0.774 (0.740–0.807)	0.830 (0.746–0.914)
10	0.778 (0.746–0.811)	0.776 (0.672–0.881)
Average	0.780	0.778

Abbreviations: AUC ROC, area under the receiver operating characteristic curve; CI, confidence interval.

### Risk-score cut points for diabetic retinopathy classification

According to the prevalence of diabetic retinopathy observed with misclassification ratios of 1, 4, and 10, the optimal cut points were −0.046, −0.640, and −1.209, respectively (Table 4).

**Table 4:** Diagnostic Tests for Cut Points of a Screening Tool for Diabetic Retinopathy for Use in Low-Income Communities, by Misclassification-Cost Ratio and Various Scenarios of Diabetic Retinopathy Prevalence, Mexico, 2014–2016

Misclassification Cost Ratio^b^	Predictive Probit Model (n = 939)^a^				
	Sensitivity, %	Specificity, %	Positive Predictive Value, %	Negative Predictive Value, %	*z* Cut Point
**Diabetic retinopathy prevalence of 31.7% (observed)**					
1	56.4	83.0	60.7	80.4	−0.046
4	82.9	61.9	50.3	88.6	−0.640
10	96.6	28.7	38.7	94.9	−1.209
**Diabetic retinopathy prevalence of 35.0%**					
1	60.1	81.1	63.2	79.1	−0.121
4	90.9	45.9	47.5	90.4	−1.017
10	96.6	28.7	42.2	94.1	−1.209
**Diabetic retinopathy prevalence of 40.0%**					
1	67.8	76.4	65.7	78.1	−0.305
4	90.9	45.9	52.8	88.4	−1.017
10	96.6	28.7	47.5	92.8	−1.209
**Diabetic retinopathy prevalence of 45.0%**					
1	71.5	74.0	69.2	76.0	−0.374
4	96.0	31.7	53.5	90.6	−1.190
10	96.6	28.7	52.6	91.3	−1.209

a Multivariate probit model with any grade of diabetic retinopathy (grade 1, grade 2, or grade 3) as dependent variable according to Revised English Diabetic Eye Screening Program Grading System (25). Estimated coefficients from the multivariate probit model are shown in Table 2.

b Misclassification-cost ratio = cost of classification of false negatives divided by cost of classification of false positives. Ratios of 1, 4, and 10 were used, assuming that false-negative classification of a person receiving diabetic retinopathy screening would generate greater health costs than would a false-positive classification.

Four points minimized the misclassification costs given the ROC curve of our model (Figure). The optimized cut point according to a misclassification ratio of 4 and the diabetic retinopathy prevalence observed in our sample (31.7%) was *z* = −0.640, with a sensitivity of 82.9%, a specificity of 61.9%, a positive predictive value of 50.3%, and a negative predictive value of 88.6% (Table 4).

**Figure:**
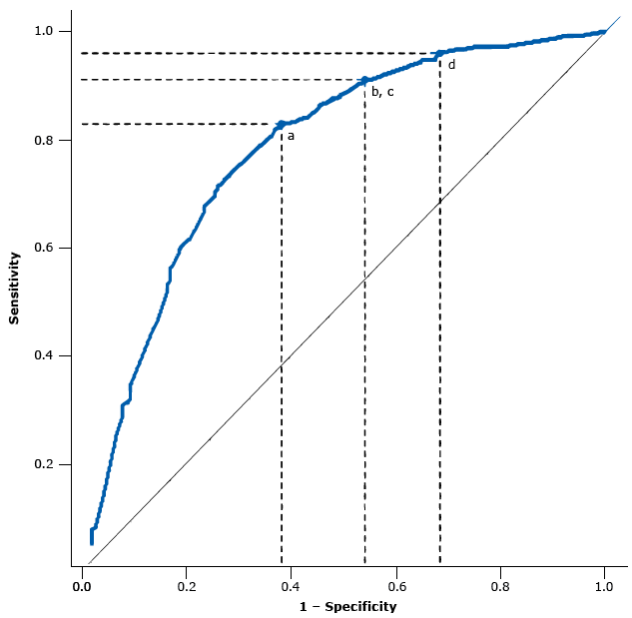
Area under the receiver operating characteristic (ROC) curve and points along the ROC curve corresponding to optimized cut points given a cost ratio (classification costs of false negatives divided by classification costs of false positives) equal to 4 and various scenarios of diabetic retinopathy prevalence: a) 31.7%, the observed prevalence in the study population; b) and c) prevalence of 35.0% and 40.0%; and d) prevalence of 45.0%. **Table:** 

Value	Point a	Point b and c	Point d
Sensitivity	0.829	0.909	0.960
1 − Specificity	0.381	0.541	0.683

On the basis of our data, we propose a risk-score screening tool (Box): A health care provider (can be a nonspecialized provider) asks the patient 2 questions (on time since diabetes diagnosis and use of physical activity to control diabetes) and obtains 2 measurements (blood glucose and systolic blood pressure). Each response is scored, the scores are summed, and a final score is calculated. The health care provider consults a simple chart that shows 4 levels of diabetic retinopathy prevalence, chooses the prevalence that most closely matches the prevalence of the community in which the patient resides, and then identifies the cut point that corresponds with the prevalence. If the patient has a score equal to or greater than the cut point, the patient should be directed to receive a comprehensive ophthalmologic evaluation.

**Box. Proposed Screening Tool for Diabetic Retinopathy in Mexican Adults Aged ≥20 With Type 2 Diabetes, Given a Cost Ratio (Classification Costs of False Negatives Divided by Classification Costs of False Positives) of 4**

**Application Instructions:**

1. Check one box per question.
2. Sum the corresponding scores of each checked box and then subtract 1.48.
3. Use the cut point closest to the diabetic retinopathy prevalence of the population in which you are applying this tool.
4. If the patient obtained a higher or equal score to the cut point used, the patient must be referred to specialized health services for a comprehensive ophthalmologic evaluation.

**Table:** 

Risk Factors for Diabetic Retinopathy	Score
**The information of the following 2 questions must be obtained by direct interview:**	
1. How long have you been diagnosed with type 2 diabetes?	
<5 years □	0
5 to 9 years □	0.55
10 to 14 years □	1.16
≥15 years □	1.41
2. Do you use physical activity to control blood sugar?	
No □	0
Yes □ (If you checked yes for this question, you must subtract 0.33)	−0.33
**The information of the following 2 questions must be obtained from measurements carried out by the interviewer: **	
3. The patient had fasting capillary or venous glucose higher or equal to 126 mg/dL or random capillary glucose higher or equal to 200 mg/dL?	
No □	0
Yes □	0.41
4. The patient presented systolic blood pressure higher or equal to 140 mm Hg?	
No □	0
Yes □	0.27
Sum of scores	
Subtract	1.48
**Final score**

- If prevalence of diabetic retinopathy is close to 31.7%, then cut point is −0.640
- If prevalence of diabetic retinopathy is close to 35.0%, then cut point is −1.017
- If prevalence of diabetic retinopathy is close to 40.0%, then cut point is −1.017
- If prevalence of diabetic retinopathy is close to 45.0%, then cut point is −1.190

## Discussion

We developed a practical screening tool for diabetic retinopathy that could be used by nonspecialized health care personnel in low-income settings. The tool requires information on 4 risk factors. Other risk scores exist (26,27); unlike these, we optimized various cut points according to misclassification costs and diabetic retinopathy prevalence. This optimization allows the application of this tool in various contexts.

We assumed that classifying people as not having diabetic retinopathy when they actually have the condition (false negative) would result in higher long-term health care costs than would classifying them with the disease when they do not have it (false positive), because without timely diagnosis and treatment, these people are likely to progress to advanced stages of the condition. We recommend using the cut points for misclassification ratios of 4 and 10, which gives greater importance to sensitivity than to specificity. Although this recommendation substantially decreases specificity, it does not imply negative health effects, because all people with type 2 diabetes should receive an ophthalmologic evaluation when diabetes is diagnosed (17).

Although the rate of false positives generated by our tool could increase health care costs (as a result of comprehensive ophthalmologic evaluations), the application of our tool could help improve compliance with recommendations for obtaining these evaluations. In addition, the benefits of timely diagnosis and treatment could compensate for any increases in health care costs.

Although we did not have complete information for a cost-benefit analysis, we showed how results changed when the relative importance of the cost of false negatives (type 2 error) to the cost of false positives (type 1 error) varied. We set false-negative rates to be higher than false-positive rates because the health care costs resulting from delays in diagnosis and treatment of false negatives may be high in the context of the screening of diabetic retinopathy. Although the classification performance of our tool was acceptable (AUC ROC > 0.75), the precision of classification depends on the false-negative rate and false-positive rate. Therefore, confirmatory assessment of all cases is mandatory. Additionally, the negative cases identified by this tool also are at some risk of diabetic retinopathy, so periodic exploratory evaluations should be performed in all patients with diabetes. 

We presented misclassification ratios only as examples: different ratios could be assumed for future research or in different contexts. Our study demonstrated a simplified approach for developing a screening tool based on a misclassification-cost criterion. Future research should focus on the assignment of costs for the 4 classification types (true positives, true negatives, false positives, and false negatives) on diabetic retinopathy screening context.

We found that systolic blood pressure and the lack of physical activity were associated with diabetic retinopathy; some studies showed that high systolic blood pressure is a potentially modifiable risk factor for diabetic retinopathy (7,12). Physical inactivity could be another important modifiable risk factor for diabetic retinopathy because it is associated with poor glycemic control (28). Our study showed that a simple question about physical activity can predict a significantly lower probability of diabetic retinopathy. Although the question cannot determine whether a person is implementing this lifestyle recommendation, it may reflect awareness and knowledge of self-care practices.

Consistent with other researchers (29,30), we observed a negative effect of obesity on diabetic retinopathy. Participants with overweight and obesity had lower levels of blood glucose and less time since diabetes diagnosis than did underweight and normal-weight participants (data not shown). We believe that the negative effect of obesity on diabetic retinopathy may be attributed to the fact that people with excess weight are experiencing an earlier stage of diabetes than people with normal or low weight.

We found a higher proportion of women (73.0%) than men in our study sample possibly because women engage in self-care practices and informal unpaid activities more than men do; this engagement may have facilitated their attendance to the recruitment process. We found a lower systolic blood pressure among women than among men (data not shown), which, given the higher proportion of women, could have underestimated the effect of systolic blood pressure in our analysis.

Our study has limitations. We did not measure HbA1c, which prevented us from adjusting our model by a variable of long-term glycemic control. However, our model adequately predicted diabetic retinopathy using parameters that are easier to measure and less expensive than an HbA1c test, which is not available at all primary health care service locations in Mexico.

An important portion of the population with type 2 diabetes may not receive a diagnosis for years (17). In Mexico, almost half of the population with diabetes is not diagnosed during routine health care, and many of them have complications that indicate many years of living with the disease (2). However, it was not possible to assess how long our study participants had been living with diabetes. Because the onset of type 2 diabetes can occur at any point during adulthood (random error), age is not the best indicator of diabetes duration. Instead of age, we used time since diagnosis as a variable for diabetes duration. Self-report of time since diabetes diagnosis may underestimate duration, but we considered it to be a nondifferential systematic error that did not affect our results. People with type 2 diabetes may recall onset of their disease inaccurately, but the inaccuracy is the same across the population of people with diabetes, and recall of onset is independent of the diabetic retinopathy condition.

The high health cost of diabetic retinopathy in Mexico is due in part to the lack of a program designed to prevent diabetes complications (15). A challenge for our team will be to develop pilot studies that evaluate the feasibility, functionality, and costs of offering our screening tool at primary health care service locations as a strategy for strengthening the system for ophthalmologic evaluation of people with diabetes.

Early detection strategies must be implemented to reduce the burden of diabetic retinopathy. Our new screening tool is a promising approach and a practical strategy with an adequate performance to detect risk of diabetic retinopathy in adults with type 2 diabetes in low-income communities in Mexico.
